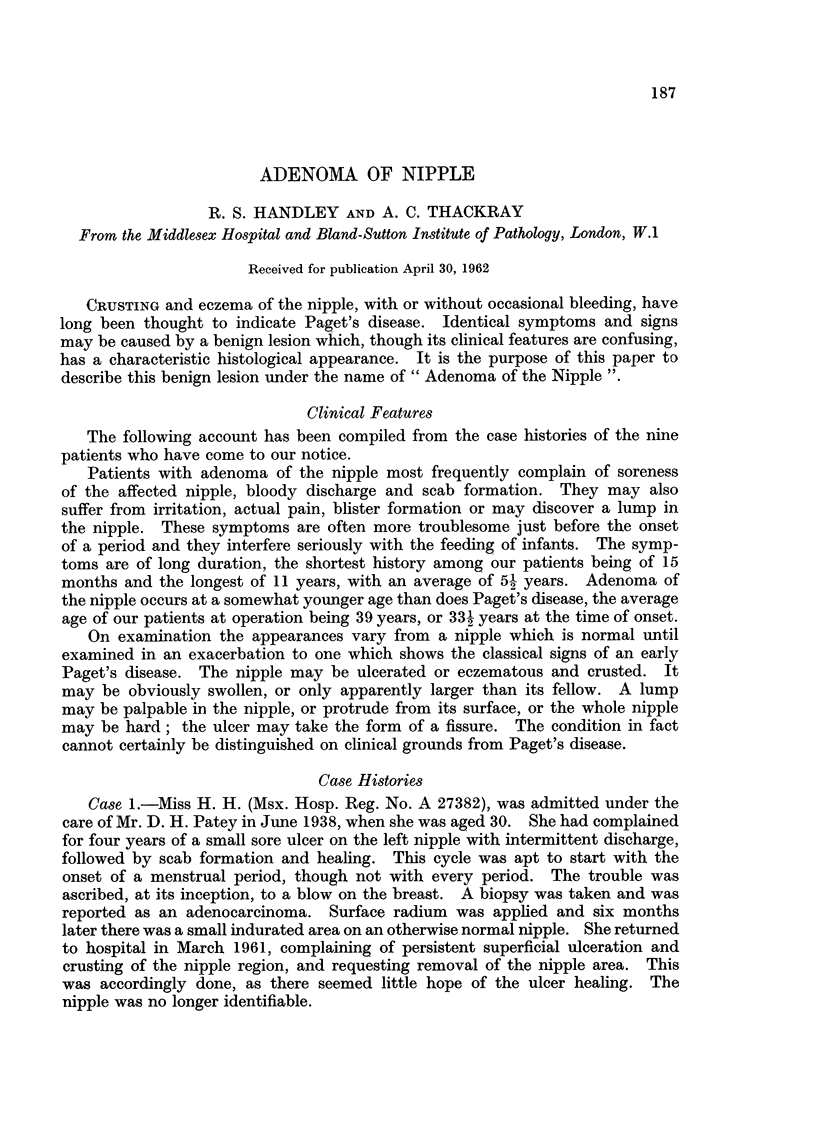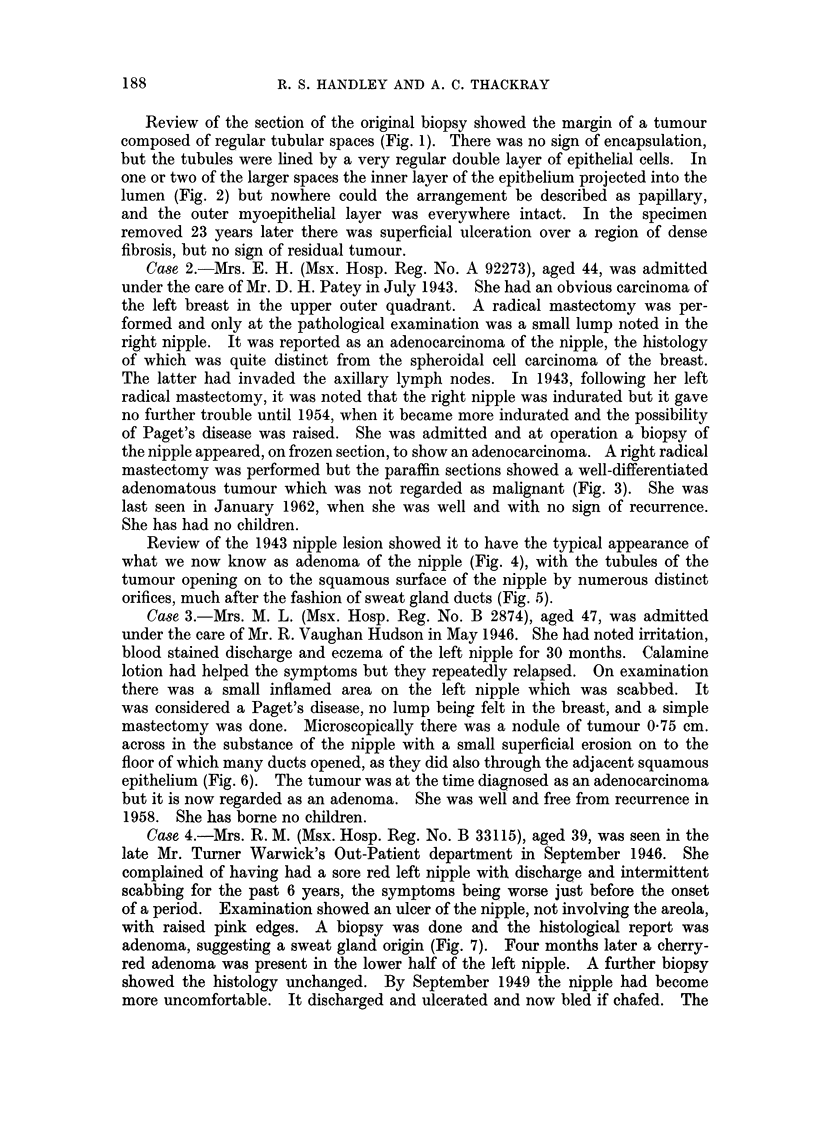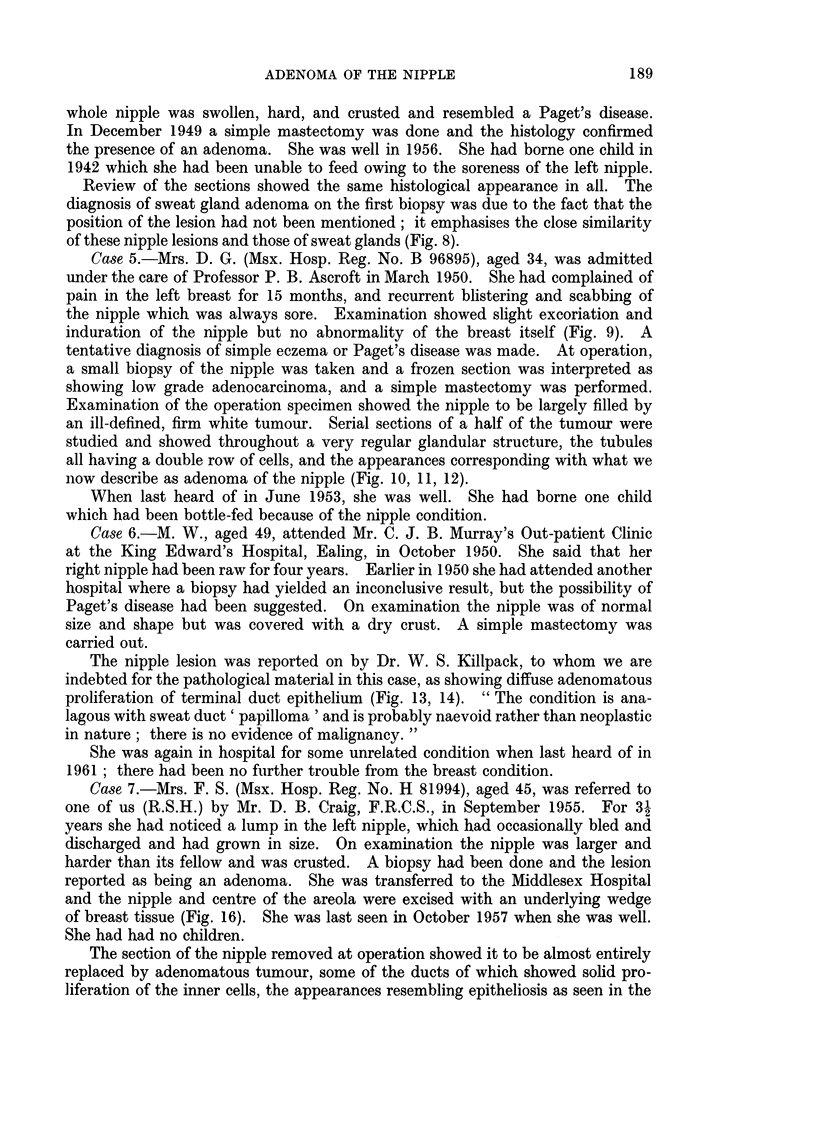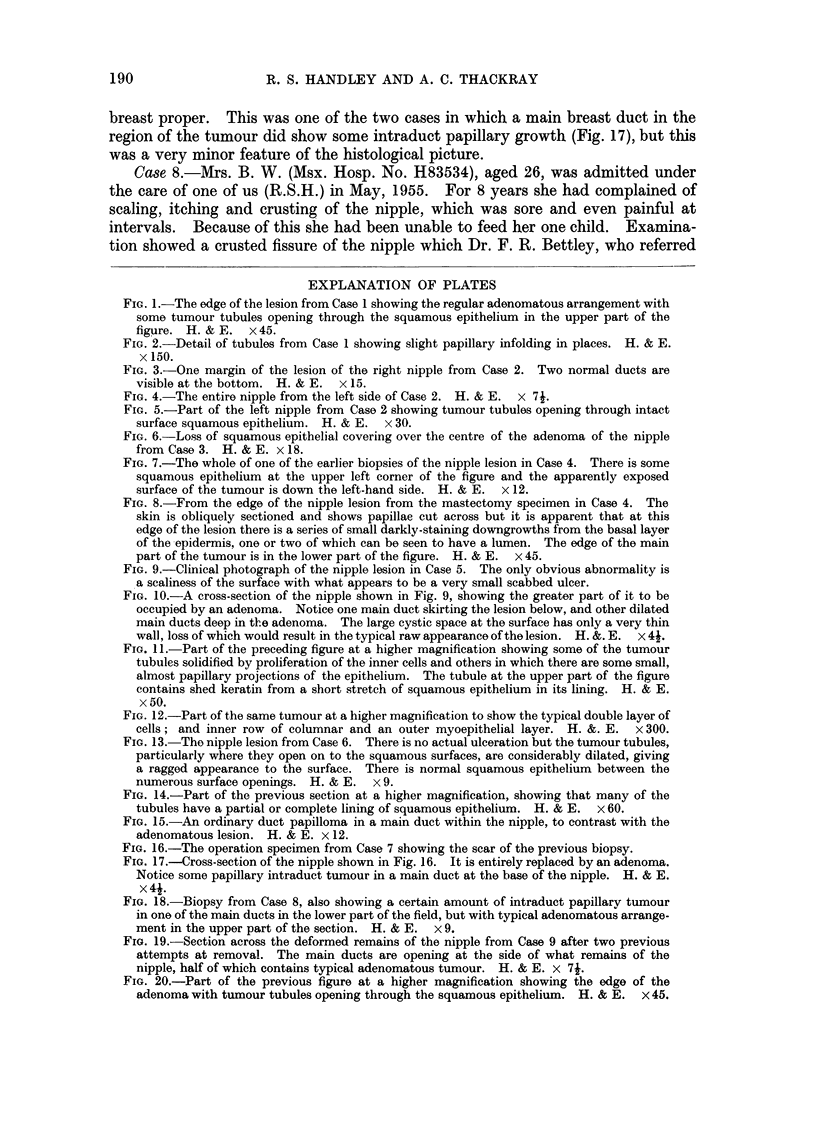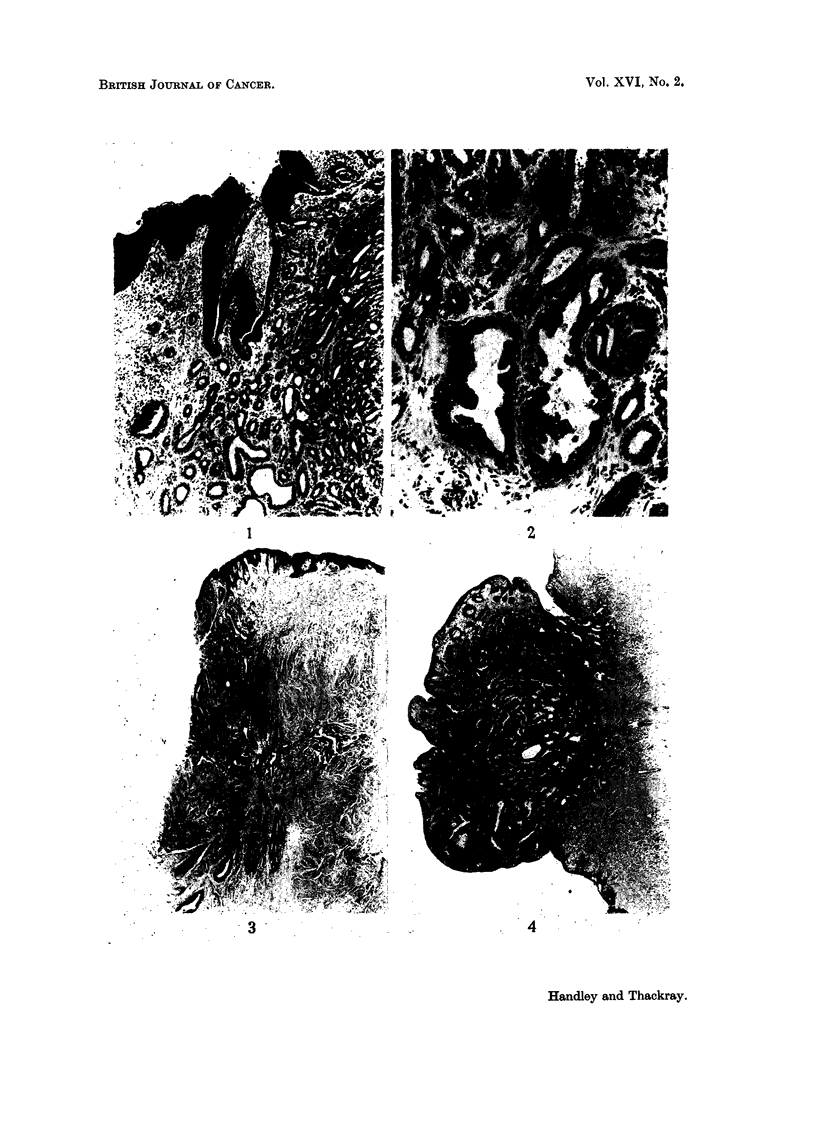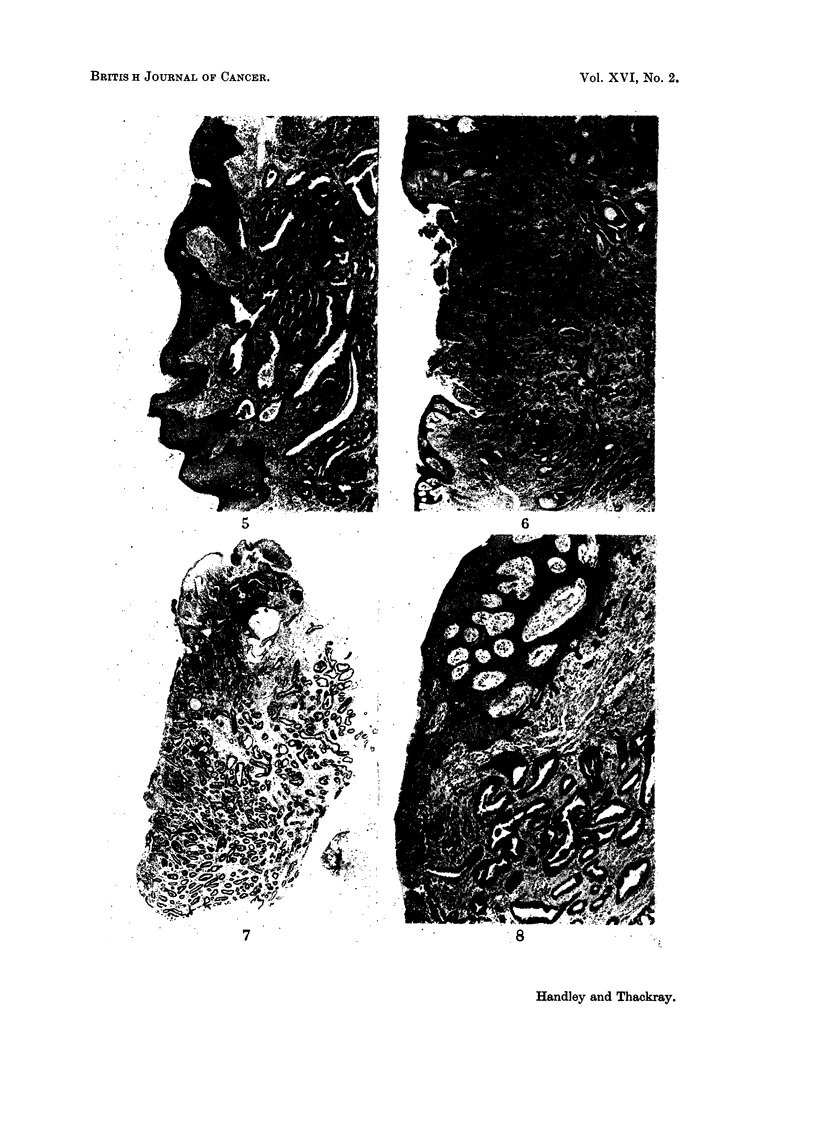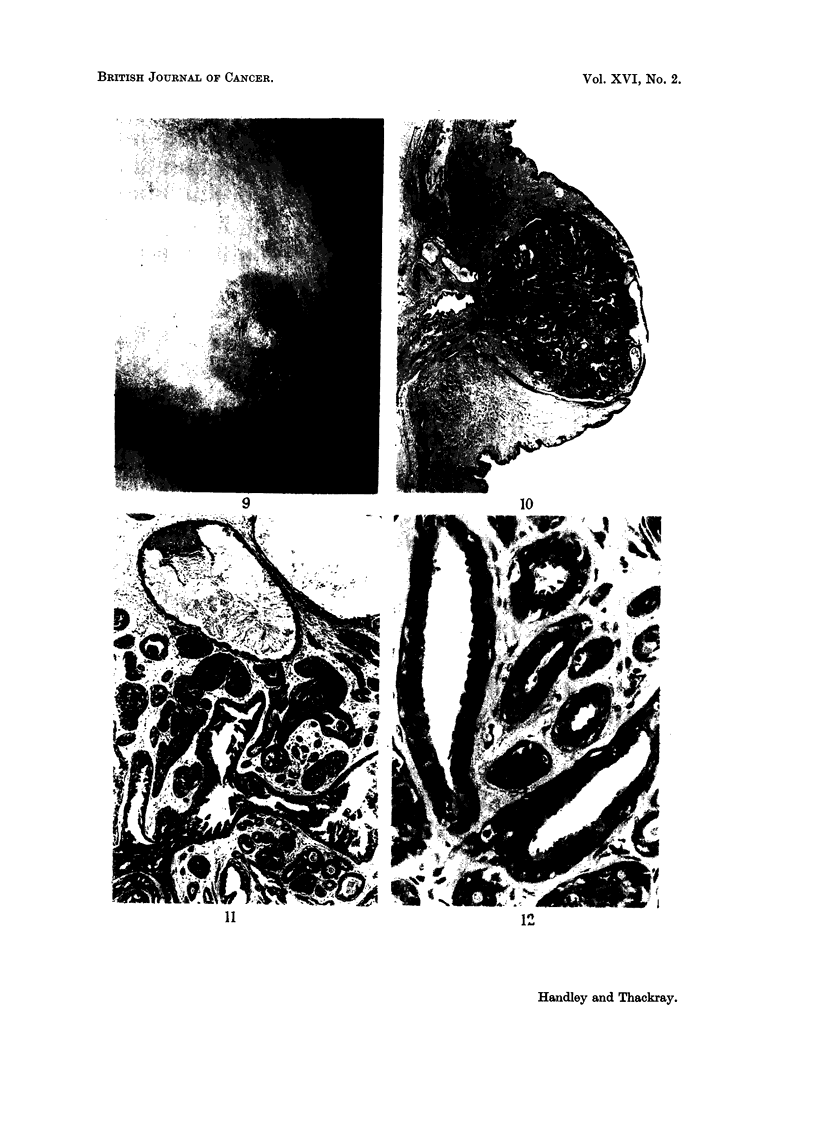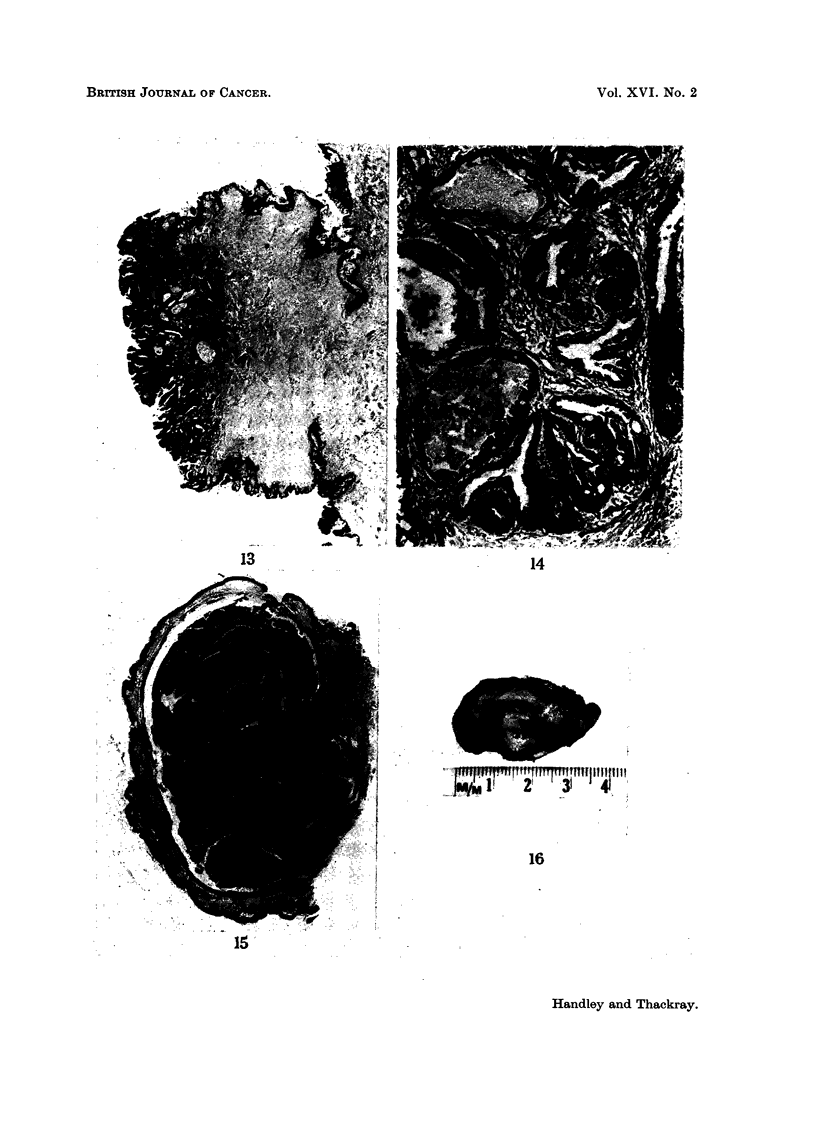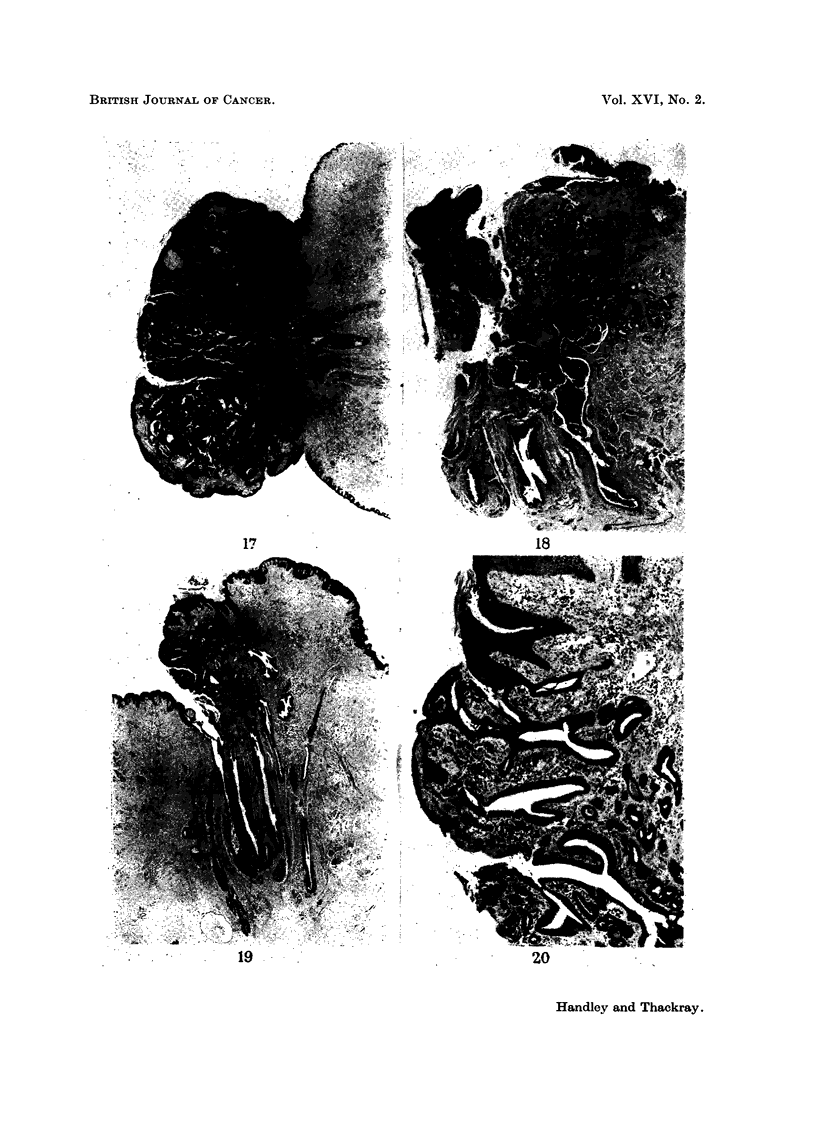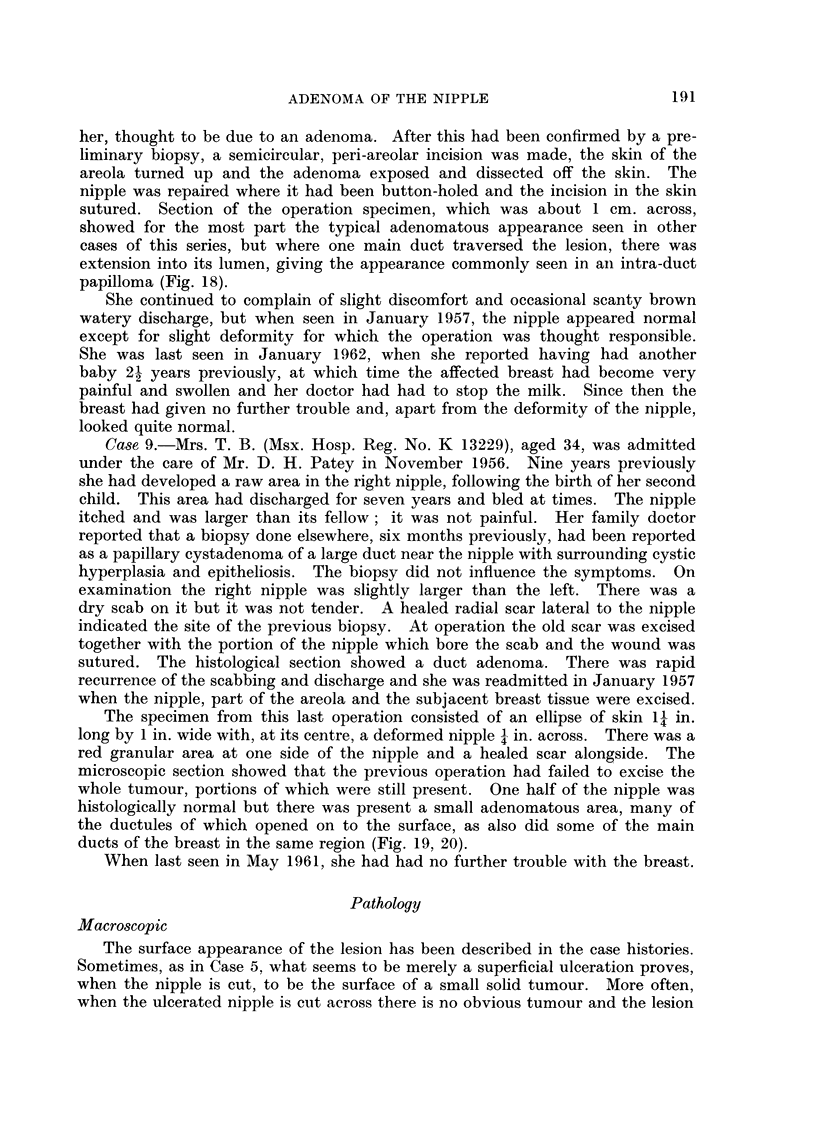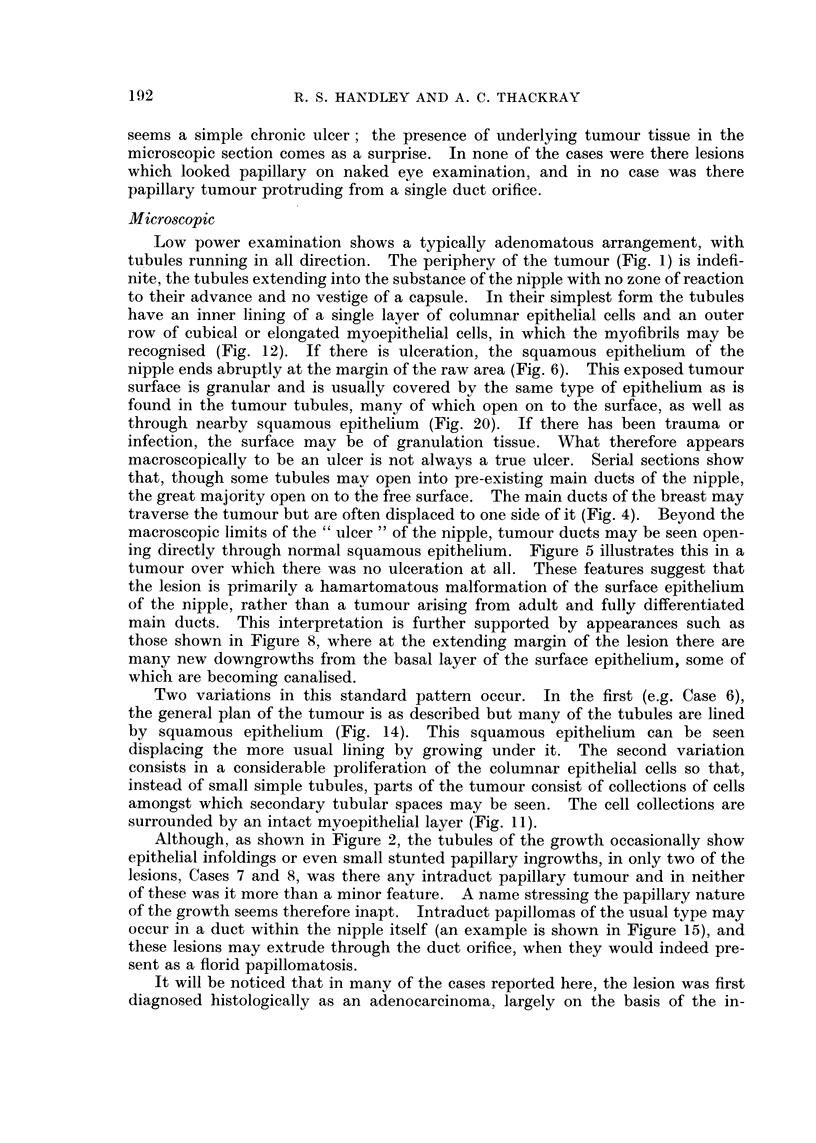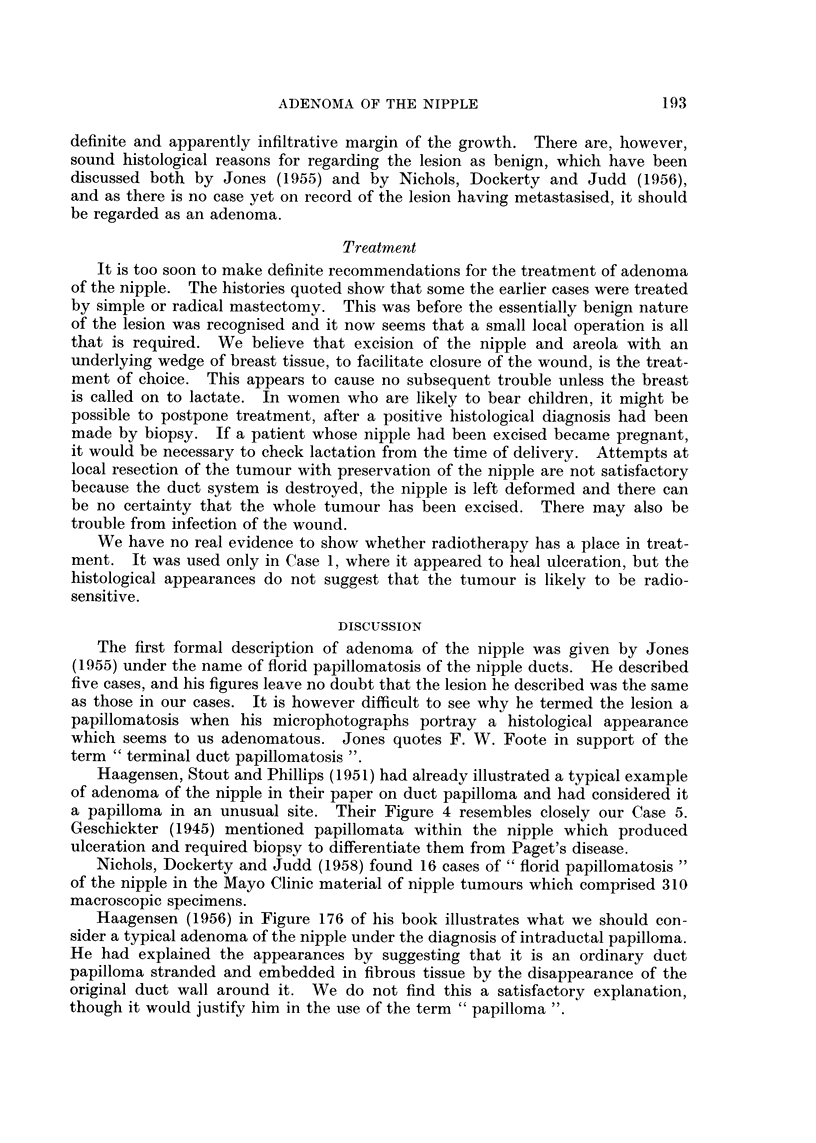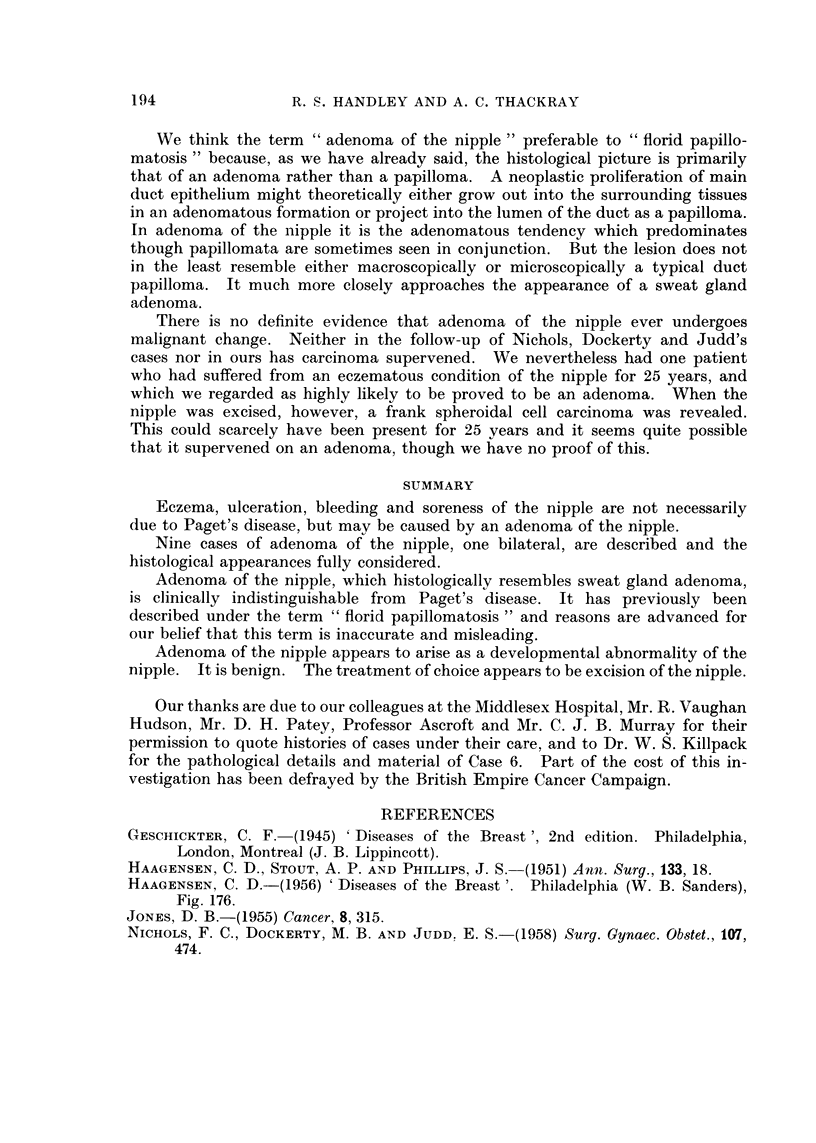# Adenoma of Nipple

**DOI:** 10.1038/bjc.1962.21

**Published:** 1962-06

**Authors:** R. S. Handley, A. C. Thackray

## Abstract

**Images:**


### ADENOMA OF NIPPLE

R. S. HANDLEY AND A. C. THACKRAY

*From the Middlesex Hospital and Bland-Sutton Institute of Pathology, London, W.1*

Received for publication April 30, 1962

CRUSTING and eczema of the nipple, with or without occasional bleeding, have long been thought to indicate Paget's disease. Identical symptoms and signs may be caused by a benign lesion which, though its clinical features are confusing, has a characteristic histological appearance. It is the purpose of this paper to describe this benign lesion under the name of " Adenoma of the Nipple ".

### *Clinical Features*

The following account has been compiled from the case histories of the nine patients who have come to our notice.

Patients with adenoma of the nipple most frequently complain of soreness of the affected nipple, bloody discharge and scab formation. They may also suffer from irritation, actual pain, blister formation or may discover a lump in the nipple. These symptoms are often more troublesome just before the onset of a period and they interfere seriously with the feeding of infants. The symptoms are of long duration, the shortest history among our patients being of 15 months and the longest of 11 years, with an average of 5½ years. Adenoma of the nipple occurs at a somewhat younger age than does Paget's disease, the average age of our patients at operation being 39 years, or 33½ years at the time of onset.

On examination the appearances vary from a nipple which is normal until examined in an exacerbation to one which shows the classical signs of an early Paget's disease. The nipple may be ulcerated or eczematous and crusted. It may be obviously swollen, or only apparently larger than its fellow. A lump may be palpable in the nipple, or protrude from its surface, or the whole nipple may be hard ; the ulcer may take the form of a fissure. The condition in fact cannot certainly be distinguished on clinical grounds from Paget's disease.

### *Case Histories*

*Case* 1.—Miss H. H. (Msx. Hosp. Reg. No. A 27382), was admitted under the care of Mr. D. H. Patey in June 1938, when she was aged 30. She had complained for four years of a small sore ulcer on the left nipple with intermittent discharge, followed by scab formation and healing. This cycle was apt to start with the onset of a menstrual period, though not with every period. The trouble was ascribed, at its inception, to a blow on the breast. A biopsy was taken and was reported as an adenocarcinoma. Surface radium was applied and six months later there was a small indurated area on an otherwise normal nipple. She returned to hospital in March 1961, complaining of persistent superficial ulceration and crusting of the nipple region, and requesting removal of the nipple area. This was accordingly done, as there seemed little hope of the ulcer healing. The nipple was no longer identifiable.

Review of the section of the original biopsy showed the margin of a tumour composed of regular tubular spaces (Fig. 1). There was no sign of encapsulation, but the tubules were lined by a very regular double layer of epithelial cells. In one or two of the larger spaces the inner layer of the epithelium projected into the lumen (Fig. 2) but nowhere could the arrangement be described as papillary, and the outer myoepithelial layer was everywhere intact. In the specimen removed 23 years later there was superficial ulceration over a region of dense fibrosis, but no sign of residual tumour.

*Case* 2.—Mrs. E. H. (Msx. Hosp. Reg. No. A 92273), aged 44, was admitted under the care of Mr. D. H. Patey in July 1943. She had an obvious carcinoma of the left breast in the upper outer quadrant. A radical mastectomy was performed and only at the pathological examination was a small lump noted in the right nipple. It was reported as an adenocarcinoma of the nipple, the histology of which was quite distinct from the spheroidal cell carcinoma of the breast. The latter had invaded the axillary lymph nodes. In 1943, following her left radical mastectomy, it was noted that the right nipple was indurated but it gave no further trouble until 1954, when it became more indurated and the possibility of Paget's disease was raised. She was admitted and at operation a biopsy of the nipple appeared, on frozen section, to show an adenocarcinoma. A right radical mastectomy was performed but the paraffin sections showed a well-differentiated adenomatous tumour which was not regarded as malignant (Fig. 3). She was last seen in January 1962, when she was well and with no sign of recurrence. She has had no children.

Review of the 1943 nipple lesion showed it to have the typical appearance of what we now know as adenoma of the nipple (Fig. 4), with the tubules of the tumour opening on to the squamous surface of the nipple by numerous distinct orifices, much after the fashion of sweat gland ducts (Fig. 5).

*Case* 3.—Mrs. M. L. (Msx. Hosp. Reg. No. B 2874), aged 47, was admitted under the care of Mr. R. Vaughan Hudson in May 1946. She had noted irritation, blood stained discharge and eczema of the left nipple for 30 months. Calamine lotion had helped the symptoms but they repeatedly relapsed. On examination there was a small inflamed area on the left nipple which was scabbed. It was considered a Paget's disease, no lump being felt in the breast, and a simple mastectomy was done. Microscopically there was a nodule of tumour 0·75 cm. across in the substance of the nipple with a small superficial erosion on to the floor of which many ducts opened, as they did also through the adjacent squamous epithelium (Fig. 6). The tumour was at the time diagnosed as an adenocarcinoma but it is now regarded as an adenoma. She was well and free from recurrence in 1958. She has borne no children.

*Case* 4.—Mrs. R. M. (Msx. Hosp. Reg. No. B 33115), aged 39, was seen in the late Mr. Turner Warwick's Out-Patient department in September 1946. She complained of having had a sore red left nipple with discharge and intermittent scabbing for the past 6 years, the symptoms being worse just before the onset of a period. Examination showed an ulcer of the nipple, not involving the areola, with raised pink edges. A biopsy was done and the histological report was adenoma, suggesting a sweat gland origin (Fig. 7). Four months later a cherry-red adenoma was present in the lower half of the left nipple. A further biopsy showed the histology unchanged. By September 1949 the nipple had become more uncomfortable. It discharged and ulcerated and now bled if chafed. The whole nipple was swollen, hard, and crusted and resembled a Paget's disease. In December 1949 a simple mastectomy was done and the histology confirmed the presence of an adenoma. She was well in 1956. She had borne one child in 1942 which she had been unable to feed owing to the soreness of the left nipple.

Review of the sections showed the same histological appearance in all. The diagnosis of sweat gland adenoma on the first biopsy was due to the fact that the position of the lesion had not been mentioned ; it emphasises the close similarity of these nipple lesions and those of sweat glands (Fig. 8).

*Case* 5.—Mrs. D. G. (Msx. Hosp. Reg. No. B 96895), aged 34, was admitted under the care of Professor P. B. Ascroft in March 1950. She had complained of pain in the left breast for 15 months, and recurrent blistering and scabbing of the nipple which was always sore. Examination showed slight excoriation and induration of the nipple but no abnormality of the breast itself (Fig. 9). A tentative diagnosis of simple eczema or Paget's disease was made. At operation, a small biopsy of the nipple was taken and a frozen section was interpreted as showing low grade adenocarcinoma, and a simple mastectomy was performed. Examination of the operation specimen showed the nipple to be largely filled by an ill-defined, firm white tumour. Serial sections of a half of the tumour were studied and showed throughout a very regular glandular structure, the tubules all having a double row of cells, and the appearances corresponding with what we now describe as adenoma of the nipple (Fig. 10, 11, 12).

When last heard of in June 1953, she was well. She had borne one child which had been bottle-fed because of the nipple condition.

*Case* 6.—M. W., aged 49, attended Mr. C. J. B. Murray's Out-patient Clinic at the King Edward's Hospital, Ealing, in October 1950. She said that her right nipple had been raw for four years. Earlier in 1950 she had attended another hospital where a biopsy had yielded an inconclusive result, but the possibility of Paget's disease had been suggested. On examination the nipple was of normal size and shape but was covered with a dry crust. A simple mastectomy was carried out.

The nipple lesion was reported on by Dr. W. S. Killpack, to whom we are indebted for the pathological material in this case, as showing diffuse adenomatous proliferation of terminal duct epithelium (Fig. 13, 14). " The condition is analagous with sweat duct ' papilloma ' and is probably naevoid rather than neoplastic in nature ; there is no evidence of malignancy. "

She was again in hospital for some unrelated condition when last heard of in 1961 ; there had been no further trouble from the breast condition.

*Case* 7.—Mrs. F. S. (Msx. Hosp. Reg. No. H 81994), aged 45, was referred to one of us (R.S.H.) by Mr. D. B. Craig, F.R.C.S., in September 1955. For 3½ years she had noticed a lump in the left nipple, which had occasionally bled and discharged and had grown in size. On examination the nipple was larger and harder than its fellow and was crusted. A biopsy had been done and the lesion reported as being an adenoma. She was transferred to the Middlesex Hospital and the nipple and centre of the areola were excised with an underlying wedge of breast tissue (Fig. 16). She was last seen in October 1957 when she was well. She had had no children.

The section of the nipple removed at operation showed it to be almost entirely replaced by adenomatous tumour, some of the ducts of which showed solid proliferation of the inner cells, the appearances resembling epitheliosis as seen in the breast proper. This was one of the two cases in which a main breast duct in the region of the tumour did show some intraduct papillary growth (Fig. 17), but this was a very minor feature of the histological picture.

*Case* 8.—Mrs. B. W. (Msx. Hosp. No. H83534), aged 26, was admitted under the care of one of us (R.S.H.) in May, 1955. For 8 years she had complained of scaling, itching and crusting of the nipple, which was sore and even painful at intervals. Because of this she had been unable to feed her one child. Examination showed a crusted fissure of the nipple which Dr. F. R. Bettley, who referred

---

EXPLANATION OF PLATES

FIG. 1.—The edge of the lesion from Case 1 showing the regular adenomatous arrangement with some tumour tubules opening through the squamous epithelium in the upper part of the figure. H. & E. ×45.

FIG. 2.—Detail of tubules from Case 1 showing slight papillary infolding in places. H. & E. ×150.

FIG. 3.—One margin of the lesion of the right nipple from Case 2. Two normal ducts are visible at the bottom. H. & E. ×15.

FIG. 4.—The entire nipple from the left side of Case 2. H. & E. × 7½.

FIG. 5.—Part of the left nipple from Case 2 showing tumour tubules opening through intact surface squamous epithelium. H. & E. ×30.

FIG. 6.—Loss of squamous epithelial covering over the centre of the adenoma of the nipple from Case 3. H. & E. ×18.

FIG. 7.—The whole of one of the earlier biopsies of the nipple lesion in Case 4. There is some squamous epithelium at the upper left corner of the figure and the apparently exposed surface of the tumour is down the left-hand side. H. & E. ×12.

FIG. 8.—From the edge of the nipple lesion from the mastectomy specimen in Case 4. The skin is obliquely sectioned and shows papillae cut across but it is apparent that at this edge of the lesion there is a series of small darkly-staining downgrowths from the basal layer of the epidermis, one or two of which can be seen to have a lumen. The edge of the main part of the tumour is in the lower part of the figure. H. & E. ×45.

FIG. 9.—Clinical photograph of the nipple lesion in Case 5. The only obvious abnormality is a scaliness of the surface with what appears to be a very small scabbed ulcer.

FIG. 10.—A cross-section of the nipple shown in Fig. 9, showing the greater part of it to be occupied by an adenoma. Notice one main duct skirting the lesion below, and other dilated main ducts deep in the adenoma. The large cystic space at the surface has only a very thin wall, loss of which would result in the typical raw appearance of the lesion. H. &. E. ×4½.

FIG. 11.—Part of the preceding figure at a higher magnification showing some of the tumour tubules solidified by proliferation of the inner cells and others in which there are some small, almost papillary projections of the epithelium. The tubule at the upper part of the figure contains shed keratin from a short stretch of squamous epithelium in its lining. H. & E. ×50.

FIG. 12.—Part of the same tumour at a higher magnification to show the typical double layer of cells; and inner row of columnar and an outer myoepithelial layer. H. &. E. ×300.

FIG. 13.—The nipple lesion from Case 6. There is no actual ulceration but the tumour tubules, particularly where they open on to the squamous surfaces, are considerably dilated, giving a ragged appearance to the surface. There is normal squamous epithelium between the numerous surface openings. H. & E. ×9.

FIG. 14.—Part of the previous section at a higher magnification, showing that many of the tubules have a partial or complete lining of squamous epithelium. H. & E. ×60.

FIG. 15.—An ordinary duct papilloma in a main duct within the nipple, to contrast with the adenomatous lesion. H. & E. ×12.

FIG. 16.—The operation specimen from Case 7 showing the scar of the previous biopsy.

FIG. 17.—Cross-section of the nipple shown in Fig. 16. It is entirely replaced by an adenoma. Notice some papillary intraduct tumour in a main duct at the base of the nipple. H. & E. ×4½.

FIG. 18.—Biopsy from Case 8, also showing a certain amount of intraduct papillary tumour in one of the main ducts in the lower part of the field, but with typical adenomatous arrangement in the upper part of the section. H. & E. ×9.

FIG. 19.—Section across the deformed remains of the nipple from Case 9 after two previous attempts at removal. The main ducts are opening at the side of what remains of the nipple, half of which contains typical adenomatous tumour. H. & E. × 7½.

FIG. 20.—Part of the previous figure at a higher magnification showing the edge of the adenoma with tumour tubules opening through the squamous epithelium. H. & E. ×45.

1

2

3

4

Handley and Thackray.

5

6

7

8

Handley and Thackray.

9

10

11

12

Handley and Thackray.

13

14

15

16

17

18

19

20

her, thought to be due to an adenoma. After this had been confirmed by a preliminary biopsy, a semicircular, peri-areolar incision was made, the skin of the areola turned up and the adenoma exposed and dissected off the skin. The nipple was repaired where it had been button-holed and the incision in the skin sutured. Section of the operation specimen, which was about 1 cm. across, showed for the most part the typical adenomatous appearance seen in other cases of this series, but where one main duct traversed the lesion, there was extension into its lumen, giving the appearance commonly seen in an intra-duct papilloma (Fig. 18).

She continued to complain of slight discomfort and occasional scanty brown watery discharge, but when seen in January 1957, the nipple appeared normal except for slight deformity for which the operation was thought responsible. She was last seen in January 1962, when she reported having had another baby $2\frac{1}{2}$ years previously, at which time the affected breast had become very painful and swollen and her doctor had had to stop the milk. Since then the breast had given no further trouble and, apart from the deformity of the nipple, looked quite normal.

*Case* 9.—Mrs. T. B. (Msx. Hosp. Reg. No. K 13229), aged 34, was admitted under the care of Mr. D. H. Patey in November 1956. Nine years previously she had developed a raw area in the right nipple, following the birth of her second child. This area had discharged for seven years and bled at times. The nipple itched and was larger than its fellow ; it was not painful. Her family doctor reported that a biopsy done elsewhere, six months previously, had been reported as a papillary cystadenoma of a large duct near the nipple with surrounding cystic hyperplasia and epitheliosis. The biopsy did not influence the symptoms. On examination the right nipple was slightly larger than the left. There was a dry scab on it but it was not tender. A healed radial scar lateral to the nipple indicated the site of the previous biopsy. At operation the old scar was excised together with the portion of the nipple which bore the scab and the wound was sutured. The histological section showed a duct adenoma. There was rapid recurrence of the scabbing and discharge and she was readmitted in January 1957 when the nipple, part of the areola and the subjacent breast tissue were excised.

The specimen from this last operation consisted of an ellipse of skin $1\frac{1}{4}$ in. long by 1 in. wide with, at its centre, a deformed nipple $\frac{1}{4}$ in. across. There was a red granular area at one side of the nipple and a healed scar alongside. The microscopic section showed that the previous operation had failed to excise the whole tumour, portions of which were still present. One half of the nipple was histologically normal but there was present a small adenomatous area, many of the ductules of which opened on to the surface, as also did some of the main ducts of the breast in the same region (Fig. 19, 20).

When last seen in May 1961, she had had no further trouble with the breast.

### *Pathology*

### *Macroscopic*

The surface appearance of the lesion has been described in the case histories. Sometimes, as in Case 5, what seems to be merely a superficial ulceration proves, when the nipple is cut, to be the surface of a small solid tumour. More often, when the ulcerated nipple is cut across there is no obvious tumour and the lesion seems a simple chronic ulcer ; the presence of underlying tumour tissue in the microscopic section comes as a surprise. In none of the cases were there lesions which looked papillary on naked eye examination, and in no case was there papillary tumour protruding from a single duct orifice.

*Microscopic*

Low power examination shows a typically adenomatous arrangement, with tubules running in all direction. The periphery of the tumour (Fig. 1) is indefinite, the tubules extending into the substance of the nipple with no zone of reaction to their advance and no vestige of a capsule. In their simplest form the tubules have an inner lining of a single layer of columnar epithelial cells and an outer row of cubical or elongated myoepithelial cells, in which the myofibrils may be recognised (Fig. 12). If there is ulceration, the squamous epithelium of the nipple ends abruptly at the margin of the raw area (Fig. 6). This exposed tumour surface is granular and is usually covered by the same type of epithelium as is found in the tumour tubules, many of which open on to the surface, as well as through nearby squamous epithelium (Fig. 20). If there has been trauma or infection, the surface may be of granulation tissue. What therefore appears macroscopically to be an ulcer is not always a true ulcer. Serial sections show that, though some tubules may open into pre-existing main ducts of the nipple, the great majority open on to the free surface. The main ducts of the breast may traverse the tumour but are often displaced to one side of it (Fig. 4). Beyond the macroscopic limits of the " ulcer " of the nipple, tumour ducts may be seen opening directly through normal squamous epithelium. Figure 5 illustrates this in a tumour over which there was no ulceration at all. These features suggest that the lesion is primarily a hamartomatous malformation of the surface epithelium of the nipple, rather than a tumour arising from adult and fully differentiated main ducts. This interpretation is further supported by appearances such as those shown in Figure 8, where at the extending margin of the lesion there are many new downgrowths from the basal layer of the surface epithelium, some of which are becoming canalised.

Two variations in this standard pattern occur. In the first (e.g. Case 6), the general plan of the tumour is as described but many of the tubules are lined by squamous epithelium (Fig. 14). This squamous epithelium can be seen displacing the more usual lining by growing under it. The second variation consists in a considerable proliferation of the columnar epithelial cells so that, instead of small simple tubules, parts of the tumour consist of collections of cells amongst which secondary tubular spaces may be seen. The cell collections are surrounded by an intact myoepithelial layer (Fig. 11).

Although, as shown in Figure 2, the tubules of the growth occasionally show epithelial infoldings or even small stunted papillary ingrowths, in only two of the lesions, Cases 7 and 8, was there any intraduct papillary tumour and in neither of these was it more than a minor feature. A name stressing the papillary nature of the growth seems therefore inapt. Intraduct papillomas of the usual type may occur in a duct within the nipple itself (an example is shown in Figure 15), and these lesions may extrude through the duct orifice, when they would indeed present as a florid papillomatosis.

It will be noticed that in many of the cases reported here, the lesion was first diagnosed histologically as an adenocarcinoma, largely on the basis of the indefinite and apparently infiltrative margin of the growth. There are, however, sound histological reasons for regarding the lesion as benign, which have been discussed both by Jones (1955) and by Nichols, Dockerty and Judd (1956), and as there is no case yet on record of the lesion having metastasised, it should be regarded as an adenoma.

### *Treatment*

It is too soon to make definite recommendations for the treatment of adenoma of the nipple. The histories quoted show that some the earlier cases were treated by simple or radical mastectomy. This was before the essentially benign nature of the lesion was recognised and it now seems that a small local operation is all that is required. We believe that excision of the nipple and areola with an underlying wedge of breast tissue, to facilitate closure of the wound, is the treatment of choice. This appears to cause no subsequent trouble unless the breast is called on to lactate. In women who are likely to bear children, it might be possible to postpone treatment, after a positive histological diagnosis had been made by biopsy. If a patient whose nipple had been excised became pregnant, it would be necessary to check lactation from the time of delivery. Attempts at local resection of the tumour with preservation of the nipple are not satisfactory because the duct system is destroyed, the nipple is left deformed and there can be no certainty that the whole tumour has been excised. There may also be trouble from infection of the wound.

We have no real evidence to show whether radiotherapy has a place in treatment. It was used only in Case 1, where it appeared to heal ulceration, but the histological appearances do not suggest that the tumour is likely to be radiosensitive.

### DISCUSSION

The first formal description of adenoma of the nipple was given by Jones (1955) under the name of florid papillomatosis of the nipple ducts. He described five cases, and his figures leave no doubt that the lesion he described was the same as those in our cases. It is however difficult to see why he termed the lesion a papillomatosis when his microphotographs portray a histological appearance which seems to us adenomatous. Jones quotes F. W. Foote in support of the term " terminal duct papillomatosis ".

Haagensen, Stout and Phillips (1951) had already illustrated a typical example of adenoma of the nipple in their paper on duct papilloma and had considered it a papilloma in an unusual site. Their Figure 4 resembles closely our Case 5. Geschickter (1945) mentioned papillomata within the nipple which produced ulceration and required biopsy to differentiate them from Paget's disease.

Nichols, Dockerty and Judd (1958) found 16 cases of " florid papillomatosis " of the nipple in the Mayo Clinic material of nipple tumours which comprised 310 macroscopic specimens.

Haagensen (1956) in Figure 176 of his book illustrates what we should consider a typical adenoma of the nipple under the diagnosis of intraductal papilloma. He had explained the appearances by suggesting that it is an ordinary duct papilloma stranded and embedded in fibrous tissue by the disappearance of the original duct wall around it. We do not find this a satisfactory explanation, though it would justify him in the use of the term " papilloma ".

We think the term " adenoma of the nipple " preferable to " florid papillomatosis " because, as we have already said, the histological picture is primarily that of an adenoma rather than a papilloma. A neoplastic proliferation of main duct epithelium might theoretically either grow out into the surrounding tissues in an adenomatous formation or project into the lumen of the duct as a papilloma. In adenoma of the nipple it is the adenomatous tendency which predominates though papillomata are sometimes seen in conjunction. But the lesion does not in the least resemble either macroscopically or microscopically a typical duct papilloma. It much more closely approaches the appearance of a sweat gland adenoma.

There is no definite evidence that adenoma of the nipple ever undergoes malignant change. Neither in the follow-up of Nichols, Dockerty and Judd's cases nor in ours has carcinoma supervened. We nevertheless had one patient who had suffered from an eczematous condition of the nipple for 25 years, and which we regarded as highly likely to be proved to be an adenoma. When the nipple was excised, however, a frank spheroidal cell carcinoma was revealed. This could scarcely have been present for 25 years and it seems quite possible that it supervened on an adenoma, though we have no proof of this.

### SUMMARY

Eczema, ulceration, bleeding and soreness of the nipple are not necessarily due to Paget's disease, but may be caused by an adenoma of the nipple.

Nine cases of adenoma of the nipple, one bilateral, are described and the histological appearances fully considered.

Adenoma of the nipple, which histologically resembles sweat gland adenoma, is clinically indistinguishable from Paget's disease. It has previously been described under the term " florid papillomatosis " and reasons are advanced for our belief that this term is inaccurate and misleading.

Adenoma of the nipple appears to arise as a developmental abnormality of the nipple. It is benign. The treatment of choice appears to be excision of the nipple.

Our thanks are due to our colleagues at the Middlesex Hospital, Mr. R. Vaughan Hudson, Mr. D. H. Patey, Professor Ascroft and Mr. C. J. B. Murray for their permission to quote histories of cases under their care, and to Dr. W. S. Killpack for the pathological details and material of Case 6. Part of the cost of this investigation has been defrayed by the British Empire Cancer Campaign.

### REFERENCES

Geschickter, C. F.—(1945) 'Diseases of the Breast', 2nd edition. Philadelphia, London, Montreal (J. B. Lippincott).

Haagensen, C. D., Stout, A. P. and Phillips, J. S.—(1951) *Ann. Surg.*, **133**, 18.

Haagensen, C. D.—(1956) 'Diseases of the Breast'. Philadelphia (W. B. Sanders), Fig. 176.

Jones, D. B.—(1955) *Cancer*, **8**, 315.

Nichols, F. C., Dockerty, M. B. and Judd, E. S.—(1958) *Surg. Gynaec. Obstet.*, **107**, 474.